# TRANSVERSE MYELITIS IN PEDIATRICS: A CASE STUDY

**DOI:** 10.51894/001c.122922

**Published:** 2024-08-31

**Authors:** Nathan Fleischman

**Affiliations:** 1 Emergency Medicine Sparrow Hospital

## 49

### INTRODUCTION

Transverse myelitis is a focal inflammatory condition of the spinal cord leading to various neurologic deficits at and/or below the level of the lesion. This is a case report of a patient who presented with neurologic deficits without obvious cause. Initial MRI studies of the spinal cord were negative, however, repeat studies several days later revealed focal lesions affecting the thoracic spine.

### CASE DESCRIPTION

A 14-year-old male presented to the ED with sudden onset RLQ pain along with chest and bilateral lower extremity numbness. He denied any recent illness or sick contacts, though he did endorse mosquito bites several days prior. Upon initial evaluation, he was found to have marked neurologic deficits of his lower extremities, including absent reflex, severely decreased strength, and loss of sensation. The patient was admitted to the PICU where he underwent extensive testing — MRI of the abdomen, brain, T- and L-spine, lumbar puncture, laboratory studies — all of which were within normal limits. The patient was started on IVIG and high-dose steroids. Then, 3 days later, the patient had a repeat MRI of the T- and L-spine, which showed hyperdense signal abnormalities affecting the spinal cord extending from the T3-T8 region, consistent with transverse myelitis. The patient required eight total days of hospitalization, with gradual improvement. Over the coming months, his symptoms gradually improved to the point where he is now walking without difficulty.

### DISCUSSION/CONCLUSIONS

This case is interesting principally in two ways. First, this is an atypical presentation of transverse myelitis. The patient initially developed right lower quadrant pain, mimicking that of appendicitis, which was the leading differential diagnosis at time of presentation. Secondly, the patient’s initial imaging studies were read as negative despite having developed profound neurologic symptoms. He was not found to have diagnostic imaging studies until several days later. This begs the question as to whether similar presentations have been observed previously.

